# An improved nerve-sparing radical hysterectomy technique for cervical cancer using the paravesico-vaginal space as a new surgical landmark

**DOI:** 10.18632/oncotarget.19011

**Published:** 2017-07-05

**Authors:** Yuqin Zhang, Tingyan Shi, Sheng Yin, Sining Ma, Di Shi, Jun Guan, Libing Xiang, Yang Liu, Yulan Ren, Deyan Tan, Rongyu Zang

**Affiliations:** ^1^ Division of Gynecologic Oncology, Department of Obstetrics and Gynecology, Zhongshan Hospital, Fudan University, Shanghai, China; ^2^ Department of Gynecology, Tumor Bank Ovarian Cancer, European Competence Center for Ovarian Cancer, Campus Virchow Clinic, Charité Medical University of Berlin, Berlin, Germany; ^3^ Nuffield Department of Obstetrics and Gynecology, University of Oxford, Oxford, United Kingdom; ^4^ Department of Gynecologic Oncology, Fudan University Cancer Center, Shanghai, China; ^5^ Department of Anatomy, Shanghai Medical College, Fudan University, Shanghai, China

**Keywords:** paravesico-vaginal space, nerve-sparing radical hysterectomy, deep uterine vein, terminal ureter, cervical cancer

## Abstract

Bladder dysfunction remains a major postoperative challenge for early stage cervical cancer patients. The present prospective phase 2 trial in patients with stage IB1 and IIA1 cervical cancer follows up on our previous, unpublished work describing a new surgical landmark, the paravesico-vaginal space. We describe a novel nerve-sparing radical hysterectomy (NSRH) approach to treat early stage cervical cancer without compromising local control rate or survival. Between September 2015 and August 2016, 49 patients were enrolled to receive NSRH. The bladder catheter was routinely removed on postoperative day 4. The primary endpoints were rate of postvoid residual urine volume (PVR) ≤ 50 ml and proportion of patients with successful catheter removal (ClinicalTrials.gov Identifier: NCT02562729). Anatomically, from ventral to dorsal, the terminal ureter, deep uterine vein, and cardinal ligament were the three markers of the paravesico-vaginal space. The median operative time was 100 min, and the median blood loss was 200 ml. Thirty-four patients (69.4%) had successful catheter removal on postoperative day 4, and 17 patients (34.7%) had a PVR ≤ 50 ml. Our results suggest that by accessing the paravesico-vaginal space landmark, the bladder branch of the inferior hypogastric plexus can be completely preserved, contributing to greater NSRH efficiency without compromising outcomes for patients with early stage cervical cancer.

## INTRODUCTION

The first radical hysterectomy with lymphadenectomy for cervical cancer was performed by Wertheim in Vienna in 1898 [1, 2]. Over the next five decades, Okabayashi and Meigs improved the safety and radicality of this approach, which became the foundation of radical hysterectomy for invasive cervical cancer [3–5]. Ever since, however, only few reports described detailed procedures for complete resection of the parametrium [6, 7].

Due to the extent of parametrial or cardinal ligament transection, the classical surgical management is usually followed by urinary dysfunction because of the injury caused to the pelvic autonomic nerves [8–10]. This is of great concern, inasmuch as preservation of autonomic nerve fibers during radical hysterectomy minimizes postoperative bladder morbidity and improves the quality of life in cervical cancer patients [11–14]. In 2006, we firstly reported an evidence-based nerve-sparing radical hysterectomy (NSRH) technique in China [15, 16], and then improved this approach in our clinical practice. Between 2011 and 2014, we conducted a preliminary retrospective study to evaluate its efficiency and safety (unpublished data).

In 2007, Fujii et al. reported their NSRH procedures in detail by describing the precise anatomy of the pelvic nerves under magnification [17, 18]. In 2008, Querleu and Morrow defined NSRH as type C1 in their new classification system for radical hysterectomy [19].However, due to the complexity of the pelvic anatomy, no global uniformity was reached about NSRH surgical procedures, particularly in regards to preservation of the bladder branch of the inferior hypogastric plexus (IHP).

Through practicing on fresh cadavers and patients who desired to preserve their bladder function, we made a breakthrough finding represented by a novel clinical anatomic landmark, the paravesico-vaginal space. By incorporating this surgical landmark into radical hysterectomy, we simplified nerve-sparing procedures by achieving: i) complete preservation of the IHP without dissecting any nerve plexus; ii) precision of parametrial transection; and iii) a shorter operation time. In our earlier exploratory study using this surgical approach, we registered successful catheter removal in 75.0% and in 85.2% of patients on postoperative day 4 and day 7, respectively (unpublished data). Following these encouraging surgical and clinical outcomes, the present prospective study was carried out between 2015 and 2016 to further validate the use of the paravesico-vaginal space landmark to improve the efficiency of NSRH.

## MATERIALS AND METHODS

### Study design and participants

This prospective, phase 2 trial was conducted in the Zhongshan Hospital, Fudan University (FDUZH). Each patient provided written informed consent before participation. This study was approved by the Ethics Committee of FDUZH, and registered with ClinicalTrials.gov (NCT02562729).

Between September 6, 2015 and August 23, 2016, patients were enrolled if they met the following inclusion criteria: FIGO stage IB1 and IIA1 cervical cancer; age between 18 and 70 years; histologically confirmed primary adenocarcinoma, squamous cell carcinoma, or adenosquamous carcinoma of the uterine cervix; no neoadjuvant chemotherapy; Karnofsky performance status ≥ 70; compliance with follow-up; provision of written informed consent. The exclusion criteria were as follow: age < 18 or > 70 years; history of lower urinary tract damage or surgery; abnormal urodynamic study results; Karnofsky performance status < 70; patients with uncontrolled psychological disorders; patients unwilling or unable to comply with the protocol; prior treatment with pelvic radiotherapy. All patients were preoperatively evaluated by pelvic magnetic resonance imaging (MRI) and abdominal computed tomography (CT).

All enrolled patients received NSRH with catheter removal routinely performed on postoperative day 4. Postvoid residual urine volume (PVR) was measured by ultrasound after spontaneous voiding. The standards for recatheterization were as follow: i) patients unable to void spontaneously or showing voiding difficulty; or ii) patients with PVR > 200 ml. In these cases, the reset catheter was removed again on postoperative day 14. Patients who did not need recatheterization received subsequent testing by ultrasound until the PVR was < 100 ml or until satisfaction of micturition or sensation of bladder fullness were achieved.

### Procedures

NSRH or nerve-sparing radical trachelectomy was followed by pelvic lymphadenectomy. Crucial surgical procedures necessary to identify the paravesico-vaginal space landmark are shown in Supplementary Table 1.

Adjuvant concurrent chemoradiotherapy (CCRT) or CCRT followed by chemotherapy using cisplatin and paclitaxel was administered according to the following criteria: more than two intermediate-risk factors, including i) pathological tumor size ≥ 4 cm, ii) stromal invasion ≥ 1/2, and iii) lymphovascular space invasion (LVSI); or more than one high-risk factor, including i) lymph node metastasis, ii) parametrial invasion, and iii) positive resection margin.

### Outcomes

The primary endpoints of this study were rate of PVR ≤ 50 ml and proportion of patients in which the catheter was removed successfully on postoperative day 4.

### Statistical analysis

Sample size considerations: The unpublished results from our previous retrospective study showed that the proportion of patients with a PVR ≤ 50 ml on postoperative day 4 increased from 10% to 39.8% after receiving our modified NSRH technique incorporating the paravesico-vaginal space landmark. Considering a type I error rate of 0.05 and a power of 90%, we planned to recruit 43 patients in the current prospective phase 2 study, a sample size calculated using the PASS software (version 11.0).

Medical records were abstracted for age, body mass index (BMI), FIGO stage, histology, operative time, estimated blood loss, postoperative hospital stay, pathology, postoperative 30-day morbidity, duration of postoperative catheterization (DPC), and PVR. BMI values were classified according to the WHO criteria [21]. Statistical computing was performed with SPSS software package for Windows (Statistical Package for the Social Sciences 19.0, SPSS Inc, Chicago, IL).

### Roles of the funding source

The funding agency supporting this study had no involvement in study design, patient recruitment, data collection, data analysis, data interpretation, or writing of the report. The corresponding author had full access to all data in the study and all authors share responsibility for the decision to submit it for publication.

## RESULTS

### Baseline and patient characteristics

Between September 6, 2015, and August 23, 2016, 49 patients were enrolled in this trial and all of them underwent NSRH and pelvic lymphadenectomy. Table 1 summarizes the clinical and pathological characteristics of the patients. The median age was 53 years (range: 34–70 years). Four patients were categorized as underweight with BMI < 18.5; 31 were normal weight or at risk of overweight with BMI 18.5–24.9; ten patients were obese class I with BMI 25–29.9; and four patients were obese class II with BMI ≥ 30 [21]. Twenty-six patients (53.1%) were diagnosed with stage Ib1, and 23 (46.9%) with stage IIa1 cervical cancer. Histology revealed 47 cases (95.9%) of squamous cell carcinoma, one case of adenocarcinoma, and one case of adenosquamous carcinoma.

**Table 1:** Clinicopathological characteristics (*N* = 49)

Variable	*N* (%)
Age, median (range), years	53 (34–70)
Body mass index, (kg/m^2^)	
< 18.5	4 (8.2)
18.5–24.9	31 (63.3)
25–29.9	10 (20.4)
≥ 30	4 (8.2)
FIGO stage	
Ib1	26 (53.1)
IIa1	23 (46.9)
Histology	
Squamous cell carcinoma	47 (95.9)
Adenocarcinoma	1 (2.0)
Adenosquamous carcinoma	1 (2.0)
Operative time, median (range), min	100 (40–175)
Estimated blood loss, median (range), ml	200 (100–2200)
Postoperative hospital stay, median (range), days	7 (5–13)
Pathological tumor size	
≤ 4 cm	44 (89.8)
> 4 cm	5 (10.2)
LVSI	
Yes	24 (49.0)
No	25 (51.0)
Stromal invasion	
< 1/2	20 (40.8)
≥ 1/2	29 (59.2)
Lymph node metastasis	
Yes	10 (20.4)
No	39 (79.6)
Positive surgical margin^a^	1 (2.0)
Adjuvant treatment	
No	24 (49.0)
CCRT	20 (40.8)
CCRT followed by chemotherapy	4 (8.2)
Chemotherapy alone^b^	1 (2.0)

Abbreviations: FIGO, International Federation of Gynecology and Obstetrics; LVSI, lymph-vascular space invasion; CCRT, concurrent chemoradiation

^a^One case showed suspicious positive vaginal surgical margin according to pathological findings.

^b^One patient with LVSI underwent abdominal trachelectomy.

### Surgical anatomy of the paravesico-vaginal space

**Figure 1:**
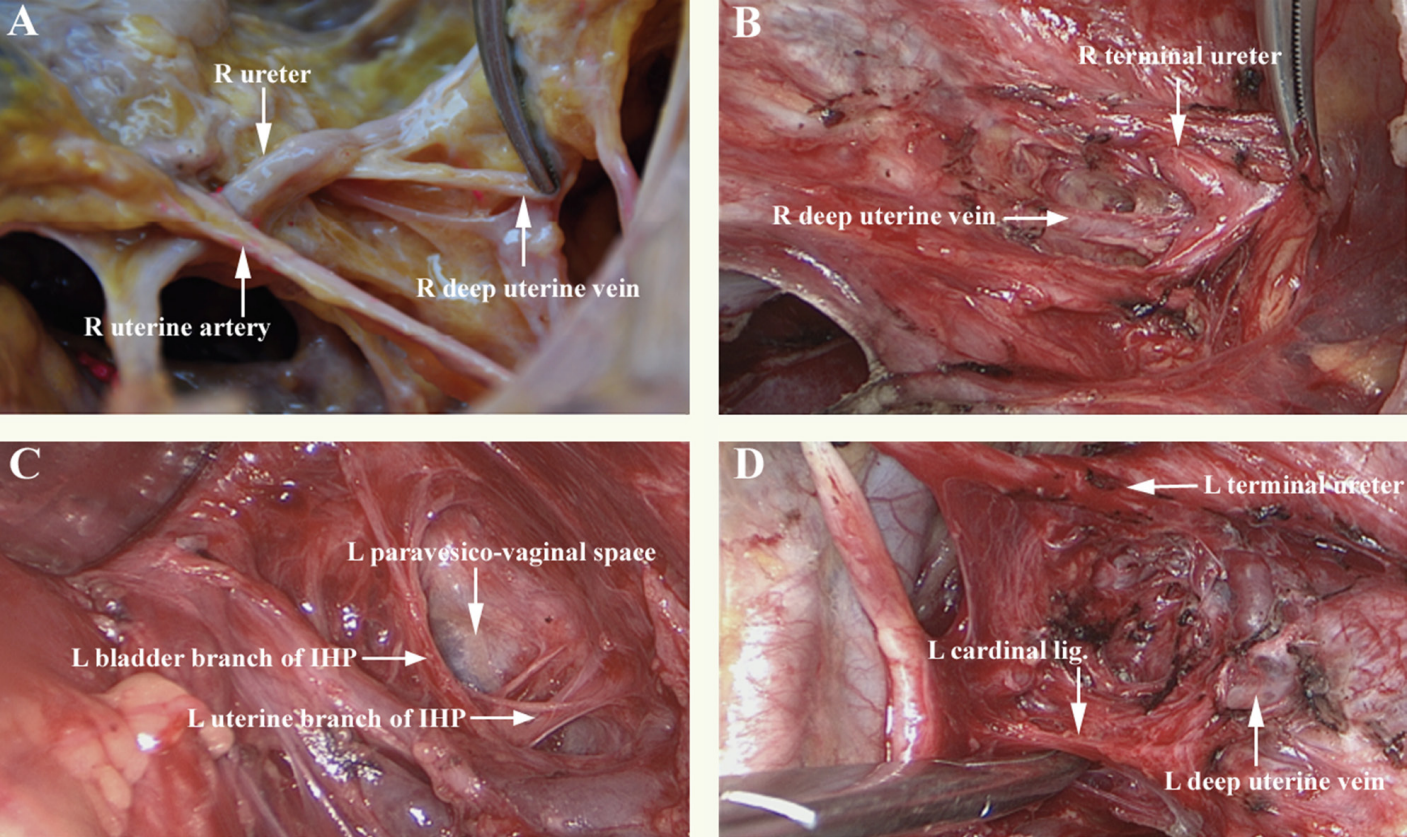
Anatomic relationship between the paravesico-vaginal space and the deep uterine vein (**A**) right, anatomical view of fresh cadavers; (**B**) right, ventral and cranial operative view (level one); (**C**) left lateral, ventral, and cranial operative view (level two); (**D**) left, operative view of the pear-shaped paravesico-vaginal space and the cardinal ligament (level three). Abbreviations: IHP, inferior hypogastric plexus; R, right; L, left.

After resecting the uterine artery, we dissected the ureteral tunnel, a free space covered by the ventral portion of the vesicouterine ligament. Then, we accessed the first layer's marker, namely the terminal ureter at the apex of the paravesico-vaginal space. On the second layer, the site of both the bladder and uterine branches of the IHP, we cut the dorsal portion of the vesicouterine ligament together with the deep uterine vein, which was the second marker. A precision sharp dissection was performed to protect all bladder branches of the IHP by pushing them laterally and caudally. Finally, the cardinal ligament was accessed at the bottom border of the paravesico-vaginal space, which was the third marker (Table 2, Figure 1, Supplementary Figure 1, and Supplementary Table 1).

**Table 2:** Anatomic hierarchy of the paravesico-vaginal space, from ventral and caudal to dorsal and cranial

Hierarchical Anatomical markers	
**LEVEL ONE**	ventral portion of the *vesicouterine ligament* *uterine artery*, *ureteral tunnel* ***terminal ureter*** (first anatomic marker)
**LEVEL TWO**	dorsal *vesicouterine ligament* ***deep uterine vein*** (second marker) *inferior hypogastric nerve plexus*
**LEVEL THREE**	paravesico-vaginal space, ***cardinal ligament*** (third marker) *inferior vesical vein*

### Surgical outcomes

As shown in Table 1, the median operative time was 100 min, ranging from 40 to 175 min. The median estimated blood loss was 200 ml (range: 100–2200 ml). The median postoperative hospital stay was 7 days, ranging from 5 to 13 days. Five patients (10.2%) presented a pathological tumor diameter > 4 cm. Twenty-four (49.0%) and 29 (59.2%) patients presented positive LVSI and deep stromal invasion, respectively. Ten patients (20.4%) presented lymph node metastasis, and one patient (2.0%) showed suspicious positive resection margin.

Primary outcomes of this study are shown in Table 3. Among the 49 patients, 34 (69.4%) had successful catheter removal on postoperative day 4, and 15 (30.6%) received recatheterization. Seventeen of 34 patients (50.0%) who underwent successful catheter removal showed a PVR ≤ 50 ml. Although three patients showed a PVR > 200 ml, all of them reported sensation of bladder filling and satisfaction of micturition. Among the 21 patients with a PVR of 50–200 ml, 14 (66.7%) had successful catheter removal on postoperative day 4 and were followed up daily by ultrasound. Thirteen patients had a PVR < 100 ml three days later, and the other one who rejected recatheterization had a PVR < 100 ml on postoperative day 14. Among the 15 patients who received recatheterization, the DPC was 14 days in 13 patients, and 20 and 21 days, respectively, in the other two.

**Table 3:** Status of bladder function recovery

Variable	*N* (%)
Catheter removal on POD 4	
Success	15 (30.6)
Failure	34 (69.4)
PVR ≤ 50 ml on POD 4	
Yes	17 (34.7)
No	32 (65.3)
Successful catheter removal on POD 4	
PVR ≤ 50 ml	17 (50.0)
50 ml < PVR ≤ 200 ml	14 (41.2)
PVR > 200 ml	3 (8.8)^a^
Catheter removal on POD 14	
Success	13 (86.7)
Failure	2 (13.3)

Abbreviations: POD, Postoperative Day; PVR, postvoid residual urine volume.

^a^All the 3 patients had sensation of bladder filling and satisfaction of micturition. The PVR was less than 100 ml on POD 7 in two patients and on POD 8 in one patient.

In total, 13 patients (26.5%) had postoperative complications according to the Common Terminology Criteria for Adverse Events (CTCAE) v4.03, but none reported with grade III–V morbidity or mortality according to the Memorial Sloan-Kettering Cancer Center (MSKCC) surgical secondary events grading system. As shown in Table 4, the most common complication was hemorrhage (*n* = 3, 6.1%), with one patient suffering an intraoperative hemorrhage with an estimated blood loss of 2200 ml. Two patients had postoperative hemorrhage according to regular hemoglobin test on peripheral blood and the volume of drainage. Other morbidities included: 2 cases of pelvic infections, 3 of urinary infections, 2 of incision infections, 1 of venous thrombosis, 1 of urinary incontinence, 1 of lymphocyst, and 1 case of lymphorrhagia. One patient suffered from both postoperative hemorrhage and pelvic infection.

**Table 4:** Assessment of complications 30 days after surgery

Complication	*N*	%
Hemorrhage	3	6.1
Intraoperative hemorrhage	1	
Postoperative hemorrhage^a^	2	
Pelvic infection	2	4.1
Urinary tract infection	3	6.1
Wound infection	2	4.1
Deep venous thrombosis	1	2.0
Urinary incontinence	1	2.0
Lymphocele	1	2.0
Lymphorrhagia	1	2.0
**MSKCC Grade III/IV**^b^	0	0
**Mortality (MSKCC Grade V)**	0	0
Total	13^c^	26.5

^a^Both patients were diagnosed with postoperative hemorrhage because of the following reasons: i) presence of bloody, continuous peritoneal drainage; ii) hemoglobin value decreased by 10 g/L in 24 hours on postoperative days 1–2.

^b^Memorial Sloan-Kettering Cancer Center surgical secondary events grading system.

^c^One case presented two complications.

## DISCUSSION

### Dissection of the ventral parametrium

Since its introduction more than 70 years ago by Meigs and Okabayashi, resection of the parametrium via the paravesical space became a widely adopted technique among gynecologists worldwide. Although commonly reported in the literature, this approach was infrequently utilized during NSRH procedures because the bladder branch of the IHP is always partly damaged by either laparotomy or laparoscopic surgery. In Fujii's study, after the development of the paravesical and pararectal space, the uterine artery and the superficial uterine vein were isolated and transected close to the pelvic sidewall [17, 18]. In another report, the paravaginal space was developed to find the dorsal vesicouterine ligament running between the paravaginal and paravesical spaces, and was mainly utilized for separating and transecting the middle and inferior vesical veins [22].

Notably, although Wertheim described in 1898 the resection of the parametrium via the medial ureter without dissecting the lateral components [1], no surgeon, to our knowledge, reported using his procedures thereafter. The current prospective study is thus the first to demonstrate that it is feasible to resect enough length of the parametrium via the inner of the terminal ureter by dividing an avascular landmark, the paravesico-vaginal space, a crucial procedure for total preservation of the bladder branch of the IHP.

### Development of the paravesico-vaginal space and its anatomic boundaries

Querleu and Morrow classified the nerve-sparing modality of radical hysterectomy as type C1 [19], but the bladder branch of the IHP was still injured during these procedures due to the uncertain relationship between the ventral parametrium and the IHP. Over the past decade, we explored and developed a more concise approach to preserve the IHP, and defined a new anatomic landmark for dissecting the ventral and medial parametrium using the modified Wertheim's method.

In contrast with most published techniques, where the deep uterine vein is used as the landmark for identification and preservation of the pelvic splanchnic nerves, the novel landmark proposed here comprises an avascular space. The paravesico-vaginal space was developed quickly and with extreme ease from the terminal ureter, after which we transected the dorsal vesicouterine ligament together with the deep uterine vein to avoid bleeding.

However, because the pelvic venous plexus, including the deep uterine vein, showed marked variability between patients, the deep uterine vein did not qualify as a stable anatomic marker. This caveat led us to explore a more stable landmark. The first and most important step was the development of the ureteral tunnel, which was completely freed by meticulous dissection of the connective tissue in the ventral portion of the vesicouterine ligament. The pear-shaped paravesico-vaginal space was delimited by the terminal ureter in its uppermost portion, the cardinal ligament at its bottom border, a bridge connecting with the deep uterine vein, and the IHP laterally.

### Comparison with previous NSRH procedures

Most previous NSRH reports include the detailed surgical procedures described by Fujii [17, 18], that clearly identify all the pelvic autonomic nerve fibers. During these procedures, vascular damage and bleeding was always inevitable, greatly increasing the difficulty to identify and preserve the nerves. The paravesico-vaginal space is an avascular, loose space amid blood vessels, nerves, and connective tissues. After incorporating this landmark, NSRH was simplified and, compared with the conventional technique, higher efficiency was achieved in terms of both surgical time and proportion of patients with PVR ≤ 50 ml on postoperative day 4. The main differences between the conventional and this novel NSRH are summarized in Supplementary Table 2.

### Efficiency and safety of NSRH

To validate the efficiency of our novel NSRH technique for early stage cervical cancer, we designed this prospective study evaluating the rate of catheter removal and PVR ≤ 50 ml on postoperative day 4 as primary end points. Successful catheter removal was accomplished in 69.4% of patients, while 34.7% showed a PVR ≤ 50 ml (Table 3). Thus, the functional efficiency of NSRH in this study was superior than that of most previous reports in the literature [13, 16, 17, 20, 22–25]. For example, in Charoenkwan's study, 21 patients received NSRH with systematic pelvic lymphadenectomy, and only 6 patients (27%) had a PVR ≤ 50 ml upon removal of the catheter on postoperative day 7 [26].

Our median operation time was 100 min. What's more, the median estimated blood loss was 200 ml. We find these results encouraging when compared to previous studies using sophisticated devices, such as the cavitron ultrasonic surgical aspirator (CUSA), laparoscopic neuro-navigation (LANN), or other techniques [22, 23, 25]. Whereas only one patient had intraoperative complications in our study, 13 (26.5%) showed some kind of morbidity by postoperative day 30. This record emphasizes the need for careful postoperative management in these patients.

In conclusion, our study introduces a novel anatomical landmark, the paravesico-vaginal space, that improves the efficacy of nerve-sparing hysterectomy. We believe our approach will help simplify conventional radical hysterectomy and decrease postoperative mortality and morbidity in patients with early stage cervical cancer.

## SUPPLEMENTARY MATERIALS FIGURES AND TABLES


