# Supplementary material for: An improved nerve-sparing radical hysterectomy technique for cervical cancer using the paravesico-vaginal space as a new surgical landmark
Source: Oncotarget. 2017 Jul 5;8(52):90413–20. doi: 10.18632/oncotarget.19011 (PMC5685761; doi:10.18632/oncotarget.19011)
Supplement: Supplementary file 1 [file oncotarget-08-90413-s001.pdf]

An improved nerve-sparing radical hysterectomy technique for cervical cancer using the paravesico-vaginal space as a new surgical landmark

Supplementary Materials

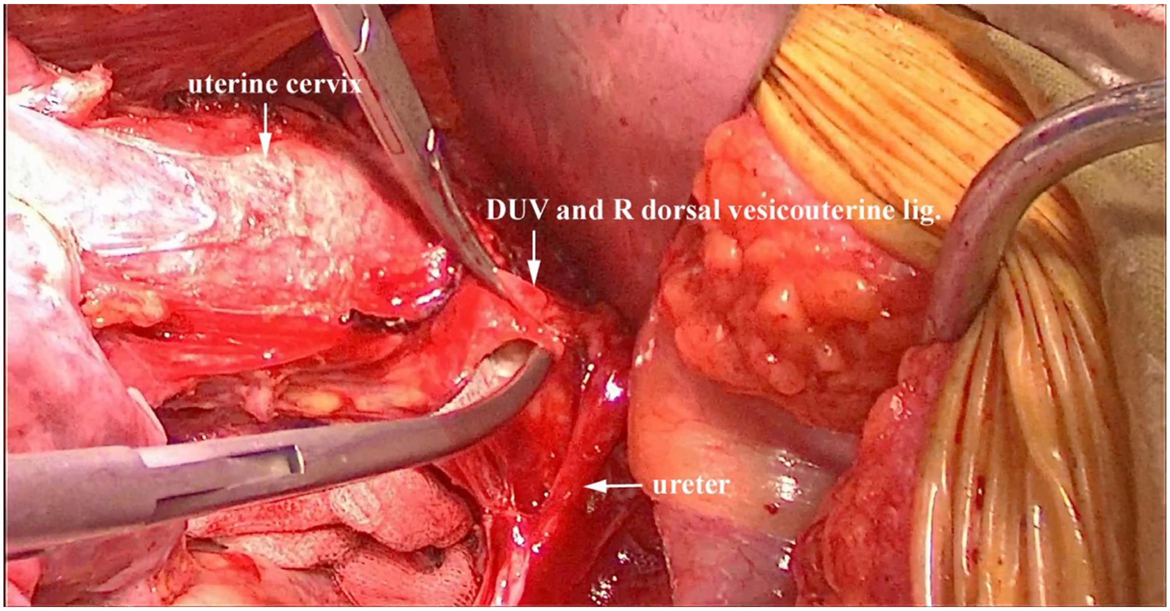

Supplementary Figure 1: A free space was found between the dorsal vesicouterine ligament (level 2) and the cardinal ligament (level 3). The deep uterine vein was then cut together with the dorsal vesicouterine ligament using a LigaSure device. Abbreviations: DUV, deep uterine vein; R, right; lig., ligament.

Supplementary Table 1: Key surgical procedures to identify the anatomical landmarks in simplified nerve-sparing radical hysterectomy

**Three crucial steps access to three anatomic landmarks**	
**1. Dissection of terminal ureter**	1.1 We used a long Kelly forceps to dissect the *ureteral tunnel*, isolating the ventral portion of *vesicouterine ligament* from the *uterine artery* to the distal ureter.<br>1.2 The *uterine artery* was isolated and cut beyond the cross of *ureter*. The *ureteral tunnel* was totally freed.<br>1.3 When the ventral portion of *vesicouterine ligament* resected, the first anatomic marker, ***terminal ureter***, was found (Figure 1B).
**2. Dissection of the deep uterine vein and the dorsal vesicouterine ligament**	2.1 After isolating the distal ureter from the ventral *vesicouterine ligament,* a tractor was applied to protect the ureter.<br>2.2 ***The deep uterine vein***, which usually ran underneath the dorsal *vesicouterine ligament*caudally and laterally, was the second anatomic landmark. We dissected the dorsal *vesicouterine ligament* together with *the deep uterine vein* using a long Kelly forceps under the vein (Figure 1C; Supplementary Figure 1).
**3. Isolation of the paravesico-vaginal space**	3.1 After resection of *the deep uterine vein,*we identified avascular-free space which was the top of the paravesico-vaginal space**.**<br>3.2 We isolated the bladder branch of *inferior hypogastricnerve plexus* to caudal and lateral, then resecting the *inferior vesico-vein.* A pear-shaped space, the paravesico-vaginal space**,** was presented together with the cardicnal ligament as Figure 1D.

Supplementary Table 2: Comparison with previous NSRH procedures

	**Previous NSRH procedures**	**Novel NSRH by *the paravesical-vaginal space***
	**Identification/Transection of Vessels**	
Middle vesical vein	In the dorsal vesicouterine ligament, by tracing the deep uterine vein from lateral to ventral direction	/
Inferior vesical vein	In the dorsal vesicouterine ligament, after separation of the deep uterine vein and the inferior vesical vein	Close to the paravesico-vaginal space
	**Identification/Dissection of Pelvic Nerves**	
The hypogastric nerve	On the rectal side of the pararectal space	/
Inferior hypogastric plexus	Identified	/
Bladder branch of IHP	Partly Preserved	Totally Preserved
